# A new era of stroke prevention in atrial fibrillation: comparing a new generation of oral anticoagulants with warfarin

**DOI:** 10.1186/1755-7682-6-46

**Published:** 2013-10-31

**Authors:** Bruce S Stambler

**Affiliations:** 1 Division of Cardiovascular Medicine, University Hospitals Case Medical Center, Case Western Reserve University, 11100 Euclid Avenue, Cleveland OH 44106, USA

**Keywords:** Atrial fibrillation, Stroke, Hemorrhage, Bleeding, Anticoagulation, Warfarin, Dabigatran, Rivaroxaban, Apixaban, Edoxaban

## Abstract

Traditionally, warfarin has been used to prevent stroke in patients with atrial fibrillation (AF), but data from large, multinational, prospective, randomized studies suggest that novel oral anticoagulants (NOACs) may be suitable alternatives. These include the direct thrombin inhibitor dabigatran and the factor Xa inhibitors rivaroxaban, apixaban, and edoxaban. These data showed that dabigatran 150 mg twice daily was more effective at preventing stroke than warfarin, with similar rates of major bleeding, while rivaroxaban 20 mg once daily was noninferior to warfarin, with no difference in major bleeding rates. In addition, apixaban 5 mg twice daily was shown to be superior to warfarin for preventing stroke, with lower bleeding rates. Currently, edoxaban is still in clinical trials. NOACs offer more predictable anticoagulant effects than warfarin and do not require regular monitoring; however, different NOACs are associated with varied drug interactions and limitations related to use in certain patient populations. Overall, the clinical data suggest that these novel agents will offer new options for stroke prevention in patients with AF.

## Introduction

Stroke is the most feared and devastating complication of atrial fibrillation (AF). On average, AF increases stroke risk 5-fold [1]. AF accounts for up to 15% of all strokes [2] and 23.5–36% of strokes in patients older than 80 years [3]. Furthermore, the long-term impact of stroke can be more serious in the presence of AF, causing higher levels of disability and greater risk of stroke recurrence and mortality than in stroke patients without AF [4-6].

The risk of stroke in patients with AF is significantly associated with specific comorbidities [7,8]. Validated moderate- to high-risk factors include mitral stenosis, a prosthetic heart valve, congestive heart failure or left ventricular ejection fraction ≤35%, hypertension, age ≥75 years, diabetes, and a history of stroke or transient ischemic attack (TIA) [3,9]. History of stroke or TIA is one of the strongest independent predictors [3,9]. Less-well-validated risk factors include female gender, vascular disease, and age 65–74 years [3]. Stroke risk increases markedly when multiple risk factors are present [9].

Anticoagulant therapy is recommended for stroke prophylaxis in patients with AF and other risk factors [3]. Chronic treatment with the vitamin K antagonist (VKA) warfarin has long been the only treatment option for reducing stroke risk in patients with AF at moderate to high risk [3]. A meta-analysis of six placebo-controlled trials showed that warfarin significantly reduced stroke risk by 64% and mortality by 26% [10].

Despite well-established supporting data, warfarin is significantly underutilized for stroke prevention in at-risk patients with AF [11]. An investigation of Medicare beneficiaries found that among 195 ideal anticoagulation candidates, only 46% were prescribed warfarin; among 111 patients aged >75 years, only 41% were prescribed warfarin [12].

A potential barrier to warfarin use is the need for regular monitoring of international normalized ratio (INR) levels to ensure they fall within the correct therapeutic range for warfarin to be effective [13,14]. However, INR levels for many patients are not consistently within this range, lowering the rate of effective warfarin use even further [15]. The need for regular monitoring results from the complicated pharmacokinetic profile of warfarin; its therapeutic window is narrow, while its pharmacokinetics are hampered by interactions with drugs, herbs, alcohol, and food, which can result in subtherapeutic or supratherapeutic drug levels [16]. Warfarin also has a slow onset of action, and initiation of treatment may require bridging anticoagulant therapy [16].

The increased risk of bleeding associated with anticoagulation further limits its use for stroke prevention in patients with AF. Use of a VKA in patients with AF increases the risk of major bleeding and intracranial hemorrhage (ICH) by 0.3–0.5% and 0.2% per year, respectively [17]. Physicians are often reluctant to prescribe warfarin to patients perceived to have a heightened risk of bleeding, such as those with a history of falls, ICH, gastrointestinal hemorrhage, or other bleeding events, even when the patient also has a coexisting very high risk of stroke [11,13]. Elderly patients with AF, who are at high risk of stroke and may derive the greatest clinical benefit from warfarin [18], are the least likely to receive warfarin, often due to perceived risks of bleeding [12].

Selection of an anticoagulant takes into consideration an assessment of its relative risks and benefits, as well as the absolute risks of stroke and bleeding for a particular patient with AF [19-22]. The limitations of warfarin highlight an unmet need in anticoagulant therapy. The landscape of pharmacologic therapy for stroke prevention in AF is currently undergoing a major revolution. Several promising novel oral anticoagulants (NOACs) have recently become available, and other agents are in development. This article describes these NOACs and reviews the clinical trial evidence for their efficacy and safety.

### Characteristics of NOACs

#### Newer anticoagulants

**Figure 1:**
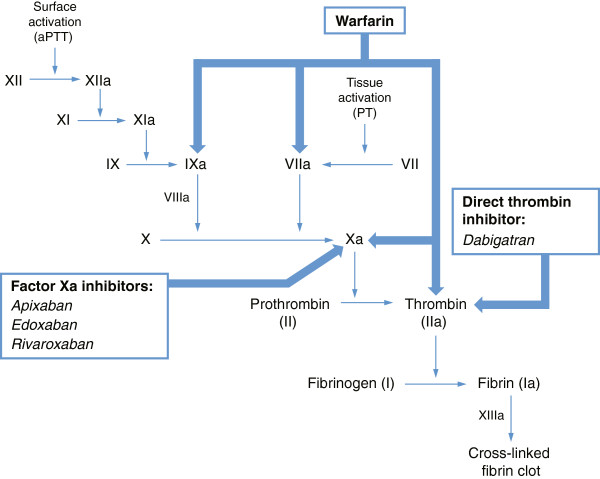
**Anticoagulant sites of action within the coagulation cascade.** Warfarin inhibits production of factors IIa, VIIa, IXa, and Xa, whereas the novel oral anticoagulants directly inhibit either the thrombin (factor IIa) molecule or factor Xa. aPTT, activated partial thromboplastin time; PT, prothrombin time.

Thrombin (factor IIa) converts fibrinogen to fibrin during the coagulation cascade, and thus plays a central role in formation of a cross-linked thrombus (clot) (Figure 1). Warfarin blocks the synthesis of multiple factors (IIa, VIIa, IXa, Xa) in the coagulation cascade to prevent thrombus formation [23]. In contrast, newer anticoagulants act specifically by inhibiting one coagulation factor [24]. Direct thrombin inhibitors (e.g. the prodrug dabigatran etexilate, which rapidly converts to the active form of dabigatran) suppress the activity of thrombin, the activated form of prothrombin (factor II), by blocking the thrombin binding site. Factor Xa inhibitors (e.g. rivaroxaban, apixaban, and edoxaban) act upstream of thrombin, reversibly inhibiting free and clot-bound factor Xa, which catalyzes the conversion of prothrombin to thrombin and therefore inhibits fibrin clot formation.

The newer anticoagulants act via direct and reversible inhibition of specific coagulation factors. They have a rapid onset and offset of action, and do not require routine monitoring of INR or other anticoagulation parameters [25]. The correlation between their plasma concentrations and coagulation measures is good, resulting in predictable anticoagulant effects [26-29]. They are metabolized primarily by the cytochrome P450 3A4/5 (CYP3A4/5) enzyme (rivaroxaban and apixaban) and/or efflux transporter P-glycoprotein (P-gp) (dabigatran and edoxaban) [27,30], which contributes to predictable pharmacokinetic responses. As a result, practitioners need to be aware of potential interactions between the NOACs and strong CYP3A or P-gp inhibitors or inducers. Bleeding risk may increase when anticoagulants are used concomitantly with antiplatelet agents including aspirin and nonsteroidal anti-inflammatory drugs. Caution should be exercised and a careful clinical assessment undertaken when NOACs and antiplatelet agents are used together.

Notably although options are under investigation, no specific reversal agents are currently available for any of the new anticoagulants. In comparison, oral or parenteral vitamin K is used for warfarin reversal, as well as prothrombin complex concentrate (PCC), fresh frozen plasma, or recombinant activated clotting factor VIIa (rFVIIa) when emergency reversal is necessary [16]. Expert guidance on the emergency reversal of the anticoagulant effects of the new agents has been published [31]. Generally, supportive care and discontinuation of therapy are recommended for patients experiencing major bleeding while on anticoagulant therapy [31]. Upon discontinuation, the anticoagulant effects of warfarin persist for 4–5 days [16]. In comparison, the NOACs have shorter half-lives and direct reversible mechanisms of action that limit their duration of action (Table 1) [32-41]. For example, for an NOAC with a half-life of approximately 12 h, the anticoagulant effect would be greatly reduced 48 h after the last dose with little, if any, effect remaining after 72 h. General reversal agents such as PCC, fresh frozen plasma, and rFVIIa might be options for counteracting NOAC anticoagulation [42]. Administration of activated charcoal may also be considered for the adsorption of unabsorbed anticoagulant and the interruption of reabsorption after biliary and/or direct intestinal secretion [33,43,44].

**Table 1:** Clinical pharmacology of novel oral anticoagulants

	**Dabigatran**[32]	**Rivaroxaban**[33]	**Apixaban**[34]	**Edoxaban**
Target of inhibition	IIa (thrombin)	Xa	Xa	Xa
Prodrug	Yes	No	No	No
Bioavailability, %	3–7	~66^a^	~50	62 [35]
Time to C_max_, h	1–2	2–4	3–4	1–2 [35,36]
Protein binding, %	35	>90	87	40–59 [36]
Substrate of P-gp drug transporter	Yes	Yes	Yes	Yes [37,38]
CYP metabolism, %	0	32	~25	Insignificant^b^[39]
Half-life, h	12–17	5–13^c^	~12	6–11^d^[36]
Renal elimination, % of total clearance	80	36 [40]	27	49 [35]
Drug interactions (examples)^e^	Strong P-gp inducers (e.g. rifampin) and inhibitors (e.g. dronedarone)	Combined P-gp and strong CYP3A4 inducers (e.g. carbamazepine) and combined P-gp and strong CYP3A4 inhibitors (e.g. ketoconazole, itraconazole)^f^	Strong inducers of both P-gp and CYP3A4 (e.g. rifampin) and strong inhibitors of both P-gp and CYP3A4 (e.g. ketoconazole)	Strong P-gp inhibitors (e.g. verapamil, quinidine) [38,41]

^a^Bioavailability of 66% in the fasted state for 20 mg dose, >66% if administered with food. ^b^Metabolites M6 and M8 are formed through CYP3A, which accounts for <4% of parent exposure. Therefore, CYP enzymes appear to have an insignificant role in the metabolism of edoxaban [39]. ^c^Five to 9 h in healthy patients aged 20–45 years, 11–13 h in elderly patients. ^d^Six to 11 h for single doses of 10–150 mg, 9–10 h for multiple doses of 60–120 mg daily [36]. ^e^Potential drug interactions that are to be avoided or may possibly require dose adjustments (not all examples are listed; see prescribing information for full details). ^f^Rivaroxaban exposure may be increased in patients with renal impairment taking P-gp and weak-to-moderate CYP3A4 inhibitors (e.g. amiodarone, diltiazem, and azithromycin).

*Abbreviations*: C_max_ maximum concentration of drug, CYP cytochrome P450, P-gp P-glycoprotein.

Temporary or permanent interruption of an anticoagulant decreases anticoagulation to subtherapeutic levels over time, increasing the risk of thromboembolic events in patients with AF. If a particular anticoagulant must be discontinued for a reason other than pathologic bleeding, use of another anticoagulant should be considered. When transitioning from one agent to another, consistent therapeutic levels should be maintained to limit the patient’s risk of thromboembolic events.

#### Dabigatran

Dabigatran etexilate mesylate (Pradaxa®) is approved as an alternative to warfarin to reduce the risk of stroke in patients with nonvalvular AF. Dabigatran is administered as a tablet twice daily [32].

Dabigatran etexilate is a prodrug that does not exhibit anticoagulant activity. After oral administration, however, it is converted rapidly by serum esterases to dabigatran, a direct thrombin inhibitor, with a resulting absolute bioavailability of approximately 3–7% [32]. Dabigatran is rapidly absorbed, reaching peak plasma levels in 1–2 h, with a half-life of between 12 and 17 h. Approximately 80% of each dose is excreted renally, with the remainder eliminated through the biliary system. This high level of renal clearance distinguishes dabigatran from warfarin and the other NOACs discussed herein. Dabigatran can be removed by dialysis, with about 60% of drug eliminated over 2–3 h [32].

In the United States, the approved dose of dabigatran is 150 mg twice daily for patients with creatinine clearance (CrCl) >30 mL/min and 75 mg twice daily for patients with CrCl 15–30 mL/min [32]. A 110-mg dose given twice daily was studied in clinical trials. This dose was not approved by the US Food and Drug Administration (FDA), but is available in Europe and Canada. The drug should not be used in patients with CrCl <15 mL/min.

Dabigatran is a substrate of the P-gp efflux transporter and has the potential to interact with inducers and inhibitors of P-gp (Table 1) [32]. Concomitant use of dabigatran with P-gp inducers (e.g. rifampin) reduces exposure to dabigatran and should be avoided [32]. Bleeding risk may rise when exposure to dabigatran is increased by way of drug interactions and diminished renal clearance. For example, dronedarone, an antiarrhythmic drug indicated for patients with AF, is a P-gp inhibitor and will increase the bioavailability of dabigatran by 1.7- to 2-fold compared with that of dabigatran given alone. A reduced dose (75 mg) is recommended when dabigatran is used in patients with CrCl 30–50 mL/min receiving the P-gp inhibitors dronedarone or systemic ketoconazole. Use of dabigatran with P-gp inhibitors is not recommended in patients with CrCl <30 mL/min [32].

#### Rivaroxaban

The reversible factor Xa inhibitor rivaroxaban (Xarelto®) is approved by the FDA for reducing the risk of stroke and systemic embolism in patients with nonvalvular AF and for treatment and prophylaxis of venous thromboembolic events in adult patients who have undergone elective hip or knee replacement surgery [33]. It is delivered orally once daily for both indications but at different doses.

The rivaroxaban 20-mg dose has a bioavailability of over 66% when taken with food (Table 1) [33]. After oral administration, rivaroxaban has a rapid onset of action, attaining peak plasma level in 2–4 h and maximum inhibition of factor Xa in 1–4 h. Half-life is 5–9 h in healthy volunteers (aged 20–45 years), but in the elderly (aged ≥65 years) it increases by up to 50% (11–13 h) [33]. At doses >5 mg, once-daily rivaroxaban administration in healthy volunteers demonstrated factor Xa inhibition through 24 h [45]. Approximately 36% of rivaroxaban is excreted renally, with the remainder metabolized in the liver and excreted into the bile for fecal elimination [33,40]. Rivaroxaban is not expected to be dialyzable due to its high plasma protein binding [46].

For stroke prophylaxis, rivaroxaban should be given once daily with the evening meal at a dose of 20 mg in patients with CrCl >50 mL/min and 15 mg in patients with CrCl 15–50 mL/min [32]. Rivaroxaban is not recommended for patients with CrCl <15 mL/min.

Drugs potentially interacting with rivaroxaban include P-gp transport and CYP450 enzyme inducers and inhibitors (Table 1) [33]. Metabolism accounted for ~51% of an orally administered dose (30% in urine and 21% in feces) [33]. Rivaroxaban is a substrate of P-gp, CYP3A4/5, CYP2J2, and adenosine triphosphate–binding cassette G2 transporter (also known as breast cancer resistance protein) [33]. Concomitant use of rivaroxaban with combined P-gp and strong CYP3A4 inducers (e.g. rifampin, carbamazepine, phenytoin, and St. John’s wort) may decrease the efficacy of rivaroxaban and should be avoided. Use with combined P-gp and strong CYP3A4 inhibitors (e.g. ketoconazole) could potentiate rivaroxaban exposure and increase bleeding risk, and thus should also be avoided [33]. Rivaroxaban exposure may also be increased in patients with renal impairment taking P-gp and weak-to-moderate CYP3A4 inhibitors (e.g. amiodarone).

#### Apixaban

Apixaban (Eliquis®) is a reversible factor Xa inhibitor approved by the FDA to reduce the risk of stroke and systemic embolism in patients with nonvalvular AF [34].

Apixaban has a bioavailability of approximately 50% for doses up to 10 mg. It is absorbed rapidly following administration, reaching peak plasma level in 3–4 h, and has a half-life of approximately 12 h [34]. Apixaban is eliminated via multiple pathways. Approximately 27% of drug is excreted renally, with the remainder excreted through hepatic metabolism as well as biliary and direct intestinal excretion. Among the NOACs currently approved by the FDA, this agent appears to have the lowest level of renal elimination [25]. Renal impairment as well as end-stage renal disease had only a modest impact on apixaban exposure, likely due to the multiple elimination pathways of apixaban [47,48].

In clinical efficacy trials, apixaban was administered at a fixed dose of 5 mg twice daily for stroke prevention. A reduced dose (2.5 mg twice daily) was used for patients who met two or more of the following criteria: ≥80 years of age, ≤60 kg body weight, and/or a serum creatinine level ≥1.5 mg/dL [49,50].

Apixaban is mainly metabolized via CYP3A4/5 and is also a substrate of P-gp transport protein and adenosine triphosphate–binding cassette G2 transporter. The apixaban dose should be decreased (2.5 mg twice daily) if used concurrently with strong dual inhibitors of CYP3A4 and P-gp, and concurrent use should be avoided if a patient is already taking a reduced dose. Concomitant use of strong dual inducers of CYP3A4 and P-gp should be avoided [34].

#### Edoxaban

Edoxaban (Lixiana®) is another investigational factor Xa inhibitor undergoing clinical development. A Phase III trial assessing edoxaban for stroke prevention in patients with AF is ongoing, and preliminary results are expected in late 2013 [51].

Edoxaban has a bioavailability of over 60%, reaches peak plasma level 1–2 h after administration, and has a half-life between 9 and 10 h (multiple daily doses of 60–120 mg) [36]. Although eliminated through multiple pathways, renal clearance represents 49% of total edoxaban clearance [35]. Exposure to edoxaban increases in patients with renal dysfunction and in individuals with low body weight (≤60 kg) [51].

Once-daily dosing with edoxaban is being investigated for the prevention of thromboembolism in patients with AF. In the Effective Anticoagulation with Factor Xa Next Generation in Atrial Fibrillation–Thrombolysis in Myocardial Infarction Study 48 (ENGAGE AF-TIMI 48), two dosing strategies are being compared with warfarin [51]. Edoxaban high and low exposure (60 and 30 mg daily) allows for dynamic dose reductions based on anticipated increased drug exposure resulting from any one or more of the following: CrCl 30–50 mL/min (Cockcroft-Gault formula), body weight ≤60 kg, or concomitant administration of verapamil or quinidine (strong P-gp inhibitors). Patients meeting any of these criteria at any time during the trial receive a 50% dose reduction (60 mg reduced to 30 mg or 30 mg reduced to 15 mg in the high- and low-exposure groups, respectively).

Edoxaban is a substrate of the P-gp efflux transporter, and edoxaban exposure increases with coadministration of the P-gp inhibitors quinidine, amiodarone, and verapamil (Table 1) [37]. CYP3A4 may also be involved in edoxaban metabolism [38,39].

### Efficacy of NOACs for stroke prevention in AF

#### NOACs versus warfarin

The designs and study populations of the clinical trials that have compared the NOACs with warfarin in reducing the risk of thromboembolism in patients with AF are summarized in Table 2[41,49,51,52], Table 3[41,49,52], and Table 4[41,49,52,53].

**Table 2:** Study designs in trials of novel oral anticoagulants versus warfarin

	**RE-LY**[41]	**ROCKET-AF**[52]	**ARISTOTLE**[49]	**ENGAGE AF-TIMI 48**[51]
Study treatments	DABI 110 mg or 150 mg BID vs WARF	RIVA 20 mg/d vs WARF	APIX 5 mg BID vs WARF	EDOX 60 mg or 30 mg/d vs WARF
Dose adjusted^a^	No	20 → 15 mg/d	5 → 2.5 mg BID	60 → 30, 30 → 15 mg/d
Design	Prospective randomized, open-label, blinded endpoint (PROBE)	Double-blind	Double-blind	Double-blind
Inclusion criteria	Nonvalvular AF in last 6 mo and ≥1 of following:	Nonvalvular AF and history of stroke, TIA, systemic embolism, or ≥2 of following:	Nonvalvular AF or AFL in last 12 mo *plus* ≥1 of following:	AF in last 12 mo and prior stroke or TIA or ≥2 of following:
	• Previous stroke or TIA	• HF or LVEF ≤35%	• Previous stroke, TIA, or systemic embolism	• History of HF
	• LVEF <40%	• Age ≥75 y	• Symptomatic HF in last 3 mo	• Age ≥75 y
	• NYHA class ≥ II HF in last 6 mo	• Hypertension	• LVEF ≤40%	• Hypertension
	• Age ≥75 y or 65–74 y *plus* diabetes, hypertension, or CAD	• Diabetes	• Age ≥75 y	• Diabetes
			• Hypertension requiring treatment	
			• Diabetes	
Main exclusion criteria	• Severe valve disease	• MS, prosthetic heart valve, LV thrombus	• Reversible AF	• Reversible AF
	• Stroke in last 14 d or severe stroke within 6 mo	• Condition that increases hemorrhage risk	• Moderate/severe MS	• Condition that increases hemorrhage risk
	• Condition that increases risk of hemorrhage	• Disabling stroke in last 3 mo or any stroke within last 14 d	• Conditions other than AF requiring anticoagulation	• Conditions other than AF requiring anticoagulation
	• CrCl <30 mL/min	• Anticoagulant therapy for condition other than AF	• Stroke within last 7 d	• Mechanical heart valve
	• Active liver disease	• Pregnancy or breast-feeding	• Need for aspirin >165 mg/d or for both aspirin and clopidogrel	• Moderate/severe MS
	• Pregnancy		• Severe renal insufficiency	• CrCl <30 mL/min
				• Dual antiplatelet therapy
				• Acute MI, stroke, ACS, or PCI in last 30 d
				• Women of childbearing potential

^a^Dose adjusted in patients with reduced drug clearance.

*Abbreviations*: ACS Acute coronary syndrome, AF Atrial fibrillation, AFL Atrial flutter, APIX Apixaban, ARISTOTLE Apixaban for Reduction in Stroke and Other Thromboembolic Events in Atrial Fibrillation, CAD Coronary artery disease, CrCl Creatinine clearance, DABI Dabigatran, EDOX Edoxaban, ENGAGE AF–TIMI 48 Effective Anticoagulation with Factor Xa Next Generation in Atrial Fibrillation–Thrombolysis in Myocardial Infarction Study 48, HF Heart failure, LV Left ventricular, LVEF Left ventricular ejection fraction, MI Myocardial infarction, MS Mitral stenosis, NYHA New York Heart Association, PCI percutaneous coronary intervention, RE-LY Randomized Evaluation of Long-Term Anticoagulation Therapy, RIVA Rivaroxaban, ROCKET-AF Rivaroxaban Once Daily Oral Direct Factor Xa Inhibition Compared with Vitamin K Antagonism for Prevention of Stroke and Embolism Trial in Atrial Fibrillation, TIA Transient ischemic attack, WARF Warfarin (dose-adjusted to international normalized ratio 2.0–3.0).

**Table 3:** Patient populations in completed trials of novel oral anticoagulants versus warfarin

	**RE-LY **[41]			**ROCKET-AF **[52]		**ARISTOTLE **[49]	
	**(n = 18,113)**			**(n = 14,264)**		**(n = 18,201)**	
	**DABI 110**	**DABI 150**	**WARF**	**RIVA**	**WARF**	**APIX**	**WARF**
Patients enrolled, n	6015	6076	6022	7131	7133	9120	9081
Age, y (SD) or (range)	71.4 (8.6)	71.5 (8.8)	71.6 (8.6)	73 (65–78)	73 (65–78)	70 (63–76)	70 (63–76)
Male patients, %	64.3	63.2	63.3	60.3	60.3	64.5	65.0
CHADS_2_ score^a^, mean (SD)	2.1 (1.1)	2.2 (1.2)	2.1 (1.1)	3.5 (0.9)	3.5 (1.0)	2.1 (1.1)	2.1 (1.1)
CHADS_2_ score^a^ ≥3, %	32.7	32.6	32.1	87.0	86.9	30.2	30.2
Prior stroke or TIA, %	19.9	20.3	19.8	54.9	54.6	19.2	19.7
Hypertension, %	78.8	78.9	78.9	90.3	90.8	87.3	87.6
Diabetes, %	23.4	23.1	23.4	40.4	39.5	25.0	24.9
Prior heart failure, %	32.2	31.8	31.9	62.6	62.3	35.5	35.4
Paroxysmal AF, %	32.1	32.6	33.8	17.5	17.8	15.1	15.5
Prior VKA use, %	50.1	50.2	48.6	62.3	62.5	57.1	57.2
Median duration of follow-up, y	2.0^b^			1.9^b^		1.8^b^	

^a^CHADS_2_ score is calculated by assigning 1 point each for a history of congestive heart failure, hypertension, age ≥75 years, or diabetes, and by assigning 2 points for a history of stroke or TIA [9].

^b^Median duration of follow-up for entire study population.

*Abbreviations:* AF, Atrial fibrillation; APIX, Apixaban; ARISTOTLE, Apixaban for Reduction in Stroke and Other Thromboembolic Events in Atrial Fibrillation; DABI, Dabigatran; RE-LY, Randomized Evaluation of Long-Term Anticoagulation Therapy; RIVA, Rivaroxaban; ROCKET-AF, Rivaroxaban Once Daily Oral Direct Factor Xa Inhibition Compared with Vitamin K Antagonism for Prevention of Stroke and Embolism Trial in Atrial Fibrillation; SD, Standard deviation; TIA, Transient ischemic attack; VKA, Vitamin K antagonist; WARF, Warfarin.

**Table 4:** Main study outcomes in trials of novel oral anticoagulants versus warfarin

	**RE-LY**[41,53]			**ROCKET-AF**[52]		**ARISTOTLE**[49]	
	**DABI 110**	**DABI 150**	**WARF**	**RIVA**	**WARF**	**APIX**	**WARF**
Stroke or systemic embolus, %/y	1.54	1.11	1.71	1.7^b^	2.2^b^	1.27	1.60
				2.1^c^	2.4^c^		
HR (95% CI)	0.65 (0.52, 0.81)^a^			0.79 (0.66, 0.96)^b^		0.79 (0.66, 0.95)	
				0.88 (0.75, 1.03)^c^			
	35			21		21	
Risk reduction, % (*P* value)	(<0.001 for noninferiority; <0.001 for superiority)^a^			(<0.001 for noninferiority)^b^; 12 (<0.001 for noninferiority; 0.12 for superiority)^c^		(<0.001 for noninferiority; 0.01 for superiority)	
Ischemic stroke, %/y	1.34	0.92	1.21	1.34^d^	1.42^d^	0.97	1.05
HR (95% CI)	0.76 (0.59, 0.97)^a^			0.94 (0.75, 1.17)		0.92 (0.74, 1.13)	
Risk reduction, % (*P* value)	24 (0.03)^a^			6 (0.581)		8 (0.42)	
Hemorrhagic stroke, %/y	0.12	0.10	0.38	0.26^d^	0.44^d^	0.24	0.47
HR (95% CI)	0.26 (0.14, 0.49)^a^			0.59 (0.37, 0.93)		0.51 (0.35, 0.75)	
Risk reduction, % (*P* value)	74 (<0.001)^a^			41 (0.024)		49 (<0.001)	
Major bleeding, %/y	2.87	3.32	3.57	3.6^d,e^	3.4^d,e^	2.13	3.09
HR (95% CI)	0.93 (0.81, 1.07)^a^			1.04 (0.90, 1.20)		0.69 (0.60, 0.80)	
Risk reduction, % (*P* value)	7 (0.31)^a^			−4 (0.58)		31 (<0.001)	
Intracranial bleeds, %/y	0.23	0.32	0.76	0.5^d^	0.7^d^	0.33	0.80
HR (95% CI)	0.41 (0.28, 0.60)^a^			0.67 (0.47, 0.93)		0.42 (0.30, 0.58)	
Risk reduction, % (*P* value)	59 (<0.001)^a^			33 (0.02)		58 (<0.001)	
Major GI bleeds, %/y	1.15	1.56	1.07	3.15^d,f^	2.16^d,f^	0.76	0.86
HR (95% CI)	1.48 (1.18, 1.85)^a^			–		0.89 (0.70, 1.15)	
Risk reduction, % (*P* value)	−52 (0.001)^a^			<0.001		11 (0.37)	
Total mortality, %/y	3.75	3.64	4.13	4.5^c^	4.9^c^	3.52	3.94
HR (95% CI)	0.88 (0.77, 1.00)^a^			0.92 (0.82, 1.03)		0.89 (0.80, 0.998)	
Risk reduction, % (*P* value)	12 (0.051)^a^			8 (0.15)		11 (0.047)	
Warfarin mean TTR, %	–	–	64	–	55	–	62

^a^Relative risk for DABI 150 mg versus WARF. ^b^Per-protocol analysis. ^c^ Intent-to-treat analysis. ^d^As-treated safety population. ^e^Any major bleeding; however, the primary safety endpoint in ROCKET-AF was combined rate of major and clinically relevant nonmajor bleeding (14.9%/y for rivaroxaban, 14.5%/y for warfarin; HR (95% CI) 1.03 (0.96, 1.11); *P* = 0.44. ^f^Proportion of patients with GI bleeding, not %/y.

*Abbreviations:* APIX apixaban, ARISTOTLE Apixaban for Reduction in Stroke and Other Thromboembolic Events in Atrial Fibrillation, CI confidence interval, DABI dabigatran, GI gastrointestinal, HR hazard ratio, RE-LY Randomized Evaluation of Long-Term Anticoagulation Therapy, RIVA rivaroxaban, ROCKET-AF Rivaroxaban Once Daily Oral Direct Factor Xa Inhibition Compared with Vitamin K Antagonism for Prevention of Stroke and Embolism Trial in Atrial Fibrillation, TTR time in therapeutic range, WARF warfarin.

#### Dabigatran

The Randomized Evaluation of Long-Term Anticoagulation Therapy (RE-LY) was a randomized, open-label, prospective trial comparing dabigatran 150 mg and 110 mg twice daily with dose-adjusted warfarin in patients with nonvalvular AF and at least one other stroke risk factor (mean CHADS_2_ score 2.1) [41]. The risk of stroke or systemic embolism was significantly lower in patients receiving dabigatran 150 mg than in those receiving warfarin (*P* < 0.001 for noninferiority; *P* < 0.001 for superiority). Stroke or systemic embolism occurred in 134 patients receiving dabigatran 150 mg (1.11% per year), 183 patients receiving dabigatran 110 mg (1.54% per year), and 202 patients receiving warfarin (1.71% per year). The 110-mg dose is not approved for use in the United States.

Major bleeding rates did not differ significantly between those receiving dabigatran 150 mg and warfarin (*P* = 0.32) [53], but gastrointestinal bleeding was significantly more frequent with dabigatran 150 mg (*P* = 0.001) [41]. Major bleeding occurred significantly less frequently with dabigatran 110 mg than with warfarin (*P* = 0.003) [53]. Fewer fatal ICHs occurred with dabigatran 150 mg (n = 13) and 110 mg (n = 11) versus warfarin (n = 32; *P* < 0.01 for both comparisons) [54]. Rates of myocardial infarction (MI) did not differ significantly for warfarin versus either dabigatran 110 mg (*P* = 0.09) or dabigatran 150 mg (*P* = 0.12), although a numerically greater risk was noted for dabigatran [53]. Annual all-cause mortality was 3.75% for dabigatran 110 mg (*P* = 0.13 vs warfarin), 3.64% for dabigatran 150 mg (*P* = 0.051 vs warfarin), and 4.13% for warfarin.

After 1 year, discontinuation rates were 15%, 16%, and 10% for dabigatran 110 mg, dabigatran 150 mg, and warfarin, respectively. These values increased to 21%, 21%, and 17% after 2 years [41]. Dyspepsia was significantly more frequent with either dose of dabigatran than with warfarin (*P* < 0.001 for both doses), and was the most common adverse event for patients receiving dabigatran.

Thromboembolic events following permanent discontinuation of dabigatran were not reported in RE-LY. It was recommended that patients being withdrawn from dabigatran be transitioned to warfarin 1–3 days prior to discontinuing dabigatran (depending on CrCl level); however, upon trial completion 48% of dabigatran-treated patients continued double-blind treatment in an extension study [55].

#### Rivaroxaban

Rivaroxaban 20 mg daily and adjusted-dose warfarin were compared in patients with nonvalvular AF in the Rivaroxaban Once Daily Oral Direct Factor Xa Inhibition Compared with Vitamin K Antagonism for Prevention of Stroke and Embolism Trial in Atrial Fibrillation (ROCKET-AF) [52]. Patients had nonvalvular AF and a history of stroke, TIA, systemic embolism, or at least two other stroke risk factors (mean CHADS_2_ score 3.5). For the primary outcome, two main analyses were performed: a per-protocol analysis including all patients who received at least one dose of study drug, had no major protocol violation, and were followed for events while on treatment or within 2 days after discontinuation; and an intent-to-treat (ITT) analysis including all randomized patients who were followed for events during treatment or after premature discontinuation. The per-protocol analysis found that stroke or systemic embolism occurred in 188 patients in the rivaroxaban group (1.7% per year) versus 241 in the warfarin group (2.2% per year) (*P* < 0.001 for noninferiority). The ITT analysis reported annual stroke or systemic embolism rates of 2.1% for rivaroxaban and 2.4% for warfarin (*P* < 0.001 for noninferiority; *P* = 0.12 for superiority).

The combined rate of the primary safety endpoint, major and clinically relevant nonmajor bleeding, did not differ significantly with rivaroxaban or warfarin (*P* = 0.44). Similarly, there was no significant difference in major bleeding between groups (3.6% vs. 3.4%, respectively; *P* = 0.58) [52]. The proportion of patients with gastrointestinal bleeding was significantly higher with rivaroxaban than with warfarin (*P* < 0.001). In the ITT population, annual all-cause mortality was 4.5% with rivaroxaban and 4.9% with warfarin (*P* = 0.15). Rates of MI did not differ between treatment groups (*P* = 0.121). Epistaxis, the most frequently observed adverse event in both treatment groups, occurred more often with rivaroxaban than with warfarin (10.14% vs. 8.55%; *P* < 0.05).

In ROCKET-AF, more strokes occurred following discontinuation of rivaroxaban than of warfarin during the 28 days after study completion (22 strokes among 4637 patients vs. 6 among 4691 patients, respectively) [33]. The protocol did not stipulate anticoagulation after study drug discontinuation. Patients discontinuing rivaroxaban were generally switched to warfarin without a period of coadministration of both drugs, which meant they may have been inadequately anticoagulated until attaining a therapeutic INR on warfarin.

#### Apixaban

The Apixaban for Reduction in Stroke and Other Thromboembolic Events in Atrial Fibrillation (ARISTOTLE) study compared the efficacy of apixaban and warfarin in nonvalvular AF [49]. In this double-blind, prospective trial, patients with nonvalvular AF or atrial flutter and at least one other stroke risk factor (mean CHADS_2_ score 2.1) were randomized to receive apixaban 5 mg twice daily or adjusted-dose warfarin. The rate of stroke or systemic embolism was significantly lower with apixaban than with warfarin (*P* < 0.001 for noninferiority; *P* = 0.01 for superiority). The primary efficacy endpoint occurred in 212 patients given apixaban (1.27% per year) compared with 265 given warfarin (1.60% per year).

Rates of major bleeding were significantly lower with apixaban than with warfarin (327 vs. 462 patients; *P* < 0.001) [49]. Annual rates of gastrointestinal bleeding were similar for apixaban and warfarin (0.76% and 0.86%, respectively; *P* = 0.37). In the ITT population, fatal bleeding (including fatal hemorrhagic stroke) occurred in 34 patients receiving apixaban and 55 receiving warfarin. All-cause mortality was significantly lower with apixaban than with warfarin (3.52% vs. 3.94%; *P* = 0.047). Rates of MI were similar between treatment groups (*P* = 0.37). Fewer patients receiving apixaban discontinued treatment (25% vs. 28% on warfarin). Adverse event rates were similar between treatment groups, and 7.6% and 8.4% of patients receiving apixaban and warfarin, respectively, discontinued treatment due to an adverse event.

Upon trial completion, a transition from the blinded study drug to open-label VKA treatment over a 2-day bridging period was recommended (85% of patients receiving apixaban started a VKA). In the 30-day period following discontinuation of apixaban there were 21 instances of stroke or systemic embolism among 6791 patients receiving apixaban who transitioned to a VKA (annual rate of 4.02%) and 5 strokes among 6569 patients receiving warfarin who continued treatment (annual rate of 0.99%). The majority of the difference in rates between groups occurred after the first week following discontinuation of apixaban [56].

### Summary of efficacy of NOACs versus warfarin

These landmark clinical trials demonstrated that dabigatran 150 mg and apixaban 5 mg twice daily were superior to warfarin, and that rivaroxaban 20 mg daily was noninferior to warfarin, in the prevention of stroke or systemic embolism in patients with nonvalvular AF [48-51]. Rates of major bleeding were similar to (dabigatran 150 mg and rivaroxaban) or lower than (dabigatran 110 mg and apixaban) those seen with warfarin. Notably, hemorrhagic stroke and ICH occurred significantly less often with all of these agents than with warfarin. Rates of gastrointestinal bleeding were significantly higher with dabigatran 150 mg and rivaroxaban, but similar with apixaban and dabigatran 110 mg compared with warfarin. Fatal bleeding was lower with rivaroxaban versus warfarin. Additionally, apixaban significantly reduced mortality in comparison with warfarin and, although nonsignificant, trends towards lower mortality were seen with dabigatran 150 mg and rivaroxaban over warfarin.

### Implications for clinical practice: a new era of anticoagulation

Warfarin has long been the standard of care for stroke prevention in patients with AF; however, after more than 60 years, a new era of stroke prophylaxis has arisen with the development of the NOACs. Data reviewed herein have shown that these new-generation oral anticoagulants are either noninferior to or more effective than warfarin for reducing the risk of stroke or systemic embolism [41,49,52].

Clinicians will likely welcome these new developments. However, having multiple agents to choose from will further complicate an already difficult decision-making process. Clinicians will have to decide not only whether the benefit versus risk favors starting, or continuing, an oral anticoagulant, but also which agent is best suited for a particular patient. Unquestionably cost will play a major role in this process; however, important differences in pharmacokinetic and pharmacodynamic properties among the various NOACs will also need to be considered (Table 1). Furthermore, trial results suggest that dose selection is critical in establishing risks/benefits, particularly for elderly patients with low body weight or impaired renal function and other markers of increased bleeding risk [21]. Clinicians will need to be mindful of drug interactions that may increase or decrease drug exposure. Fortunately these types of interactions occur less often with the NOACs than with warfarin. Comparing individual NOACs is difficult without head-to-head clinical trials. However, awareness of subtle differences among the patient populations and results of clinical trials may help clinicians in choosing the most appropriate NOAC for individual patients (see Tables 2, 3, and 4). Adherence to treatment has a critical impact on treatment efficacy and safety, but is currently difficult to monitor with the NOACs. Therefore, when selecting a treatment clinicians might also consider factors that influence adherence, such as dosing schedules, adverse events, and reasons for discontinuation (e.g. dyspepsia, minor bleeding).

Real-world postmarketing data are beginning to emerge relating to the use of NOACs for stroke prevention in patients with AF [57]. Poor renal function, patient age, and prescriber error appear to be important factors associated with bleeding episodes.

Clinical trial results, although suggesting benefits of NOACs over warfarin, also raise the question of whether patients who have stable INR levels and are well-controlled on warfarin should be switched to NOACs and, if so, what criteria should be considered when making the switch. The more predictable anticoagulant effect of the novel drugs obviates the frequent anticoagulation monitoring that warfarin treatment requires. Although ICH is a rare event with any anticoagulant, it is the most feared complication as its consequences are frequently catastrophic. The NOACs consistently reduce ICH and hemorrhagic stroke compared with warfarin, which is an important advantage. On the other hand, the lack of a specific reversal agent for these agents may limit their acceptance. The inability to monitor anticoagulation efficacy and compliance may also hamper enthusiasm for NOACs, given the long-standing experience many practitioners have with warfarin. Therefore, prescribers may initially be cautious about using these new drugs in otherwise appropriate candidates for these therapies. Furthermore, warfarin will still be used in patient subgroups in which the efficacy and safety of NOACs have not been elucidated and contrasted, including patients with mechanical heart valves or hemodynamically significant valvular heart disease, pregnant women, women of reproductive age, and children [58]. Notably, the RE-ALIGN (Randomized, phase II study to Evaluate the sAfety and pharmacokinetics of oraL dabIGatran etexilate in patients after heart valve replacemeNt) was terminated early, after enrollment of 252 patients with mechanical heart valves, because patients in the dabigatran group experienced an excess of thromboembolic and bleeding events. Thus, data will likely continue to emerge to inform the use of the NOACs in different patient subgroups. Patients with poor renal function or on dialysis cannot be treated with these novel agents. Current data are limited on the use of NOACs in procedures or populations where anticoagulation is routinely used, such as surrounding cardioversion, catheter ablation for AF or coronary artery interventions, and patients with acute coronary syndromes or a recent (<14 days) stroke.

Although challenges may continue to exist, the data suggest that the novel agents will offer new options for stroke prevention in patients with AF. Furthermore, the NOACs may offer the opportunity to effectively treat patients at risk of stroke who currently do not receive any anticoagulation.

## Conclusions

NOACs, both direct thrombin and factor Xa inhibitors, promise to be useful alternatives to warfarin for stroke prophylaxis in patients with nonvalvular AF, based on current clinical trial data. Dabigatran 150 mg twice daily is more effective than dose-adjusted warfarin, with similar rates of major bleeding, whereas rivaroxaban 20 mg once daily is noninferior to warfarin, with no difference in major bleeding. Apixaban 5 mg twice daily is more effective than warfarin, causes less bleeding, and provides a mortality benefit over warfarin. The new generation of orally administered anticoagulants will present new treatment options for clinicians managing patients with AF, and their use ultimately could lead to an overall reduction in the incidence of stroke.

## Abbreviations

AF: Atrial fibrillation; ARISTOTLE: Apixaban for Reduction in Stroke and Other Thromboembolic Events in Atrial Fibrillation; CrCl: Creatinine clearance; CYP: Cytochrome P450; FDA: Food and Drug Administration; ICH: Intracranial hemorrhage; INR: International normalized ratio; ITT: Intent-to-treat; MI: Myocardial infarction; NOAC: Novel oral anticoagulant; PCC: Prothrombin complex concentrate; P-gp: P-glycoprotein; RE-LY: Randomized Evaluation of Long-Term Anticoagulation Therapy; rFVIIa: Recombinant activated clotting factor VIIa; ROCKET-AF: Rivaroxaban Once Daily Oral Direct Factor Xa Inhibition Compared with Vitamin K Antagonism for Prevention of Stroke and Embolism Trial in Atrial Fibrillation; TIA: Transient ischemic attack; VKA: Vitamin K antagonist.

## Competing interests

Bruce S. Stambler has received honoraria and consulting fees from Bayer, Biotronik, Boehringer-Ingelheim, Boston Scientific, Bristol-Myers Squibb, CV Therapeutics, Janssen Pharmaceuticals, Johnson & Johnson, Medtronic, Pfizer, sanofi-aventis, and St Jude Medical, and independent research grants from Pfizer and CV Therapeutics.
